# Disrupted Module Efficiency of Structural and Functional Brain Connectomes in Clinically Isolated Syndrome and Multiple Sclerosis

**DOI:** 10.3389/fnhum.2018.00138

**Published:** 2018-04-10

**Authors:** Yaou Liu, Yunyun Duan, Huiqing Dong, Frederik Barkhof, Kuncheng Li, Ni Shu

**Affiliations:** ^1^Department of Radiology, Beijing Tiantan Hospital, Capital Medical University, Beijing, China; ^2^Tiantan Image Research Center, China National Clinical Research Center for Neurological Diseases, Beijing, China; ^3^Department of Radiology, Xuanwu Hospital, Capital Medical University, Beijing, China; ^4^Department of Radiology and Nuclear Medicine, VU University Medical Center, Amsterdam, Netherlands; ^5^Department of Neurology, Xuanwu Hospital, Capital Medical University, Beijing, China; ^6^Institute of Neurology and Healthcare Engineering, University College London, London, United Kingdom; ^7^State Key Laboratory of Cognitive Neuroscience and Learning and IDG/McGovern Institute for Brain Research, Beijing Normal University, Beijing, China; ^8^Center for Collaboration and Innovation in Brain and Learning Sciences, Beijing Normal University, Beijing, China; ^9^Beijing Key Laboratory of Brain Imaging and Connectomics, Beijing Normal University, Beijing, China

**Keywords:** multiple sclerosis, clinically isolated syndrome, diffusion MRI, functional MRI, graph theory, brain network

## Abstract

Recent studies have demonstrated disrupted topological organization of brain connectome in multiple sclerosis (MS). However, whether the communication efficiency between different functional systems is affected in the early stage of MS remained largely unknown. In this study, we constructed the structural connectivity (SC) and functional connectivity (FC) networks in 41 patients with clinically isolated syndrome (CIS), 32 MS patients and 35 healthy controls (HC) based on diffusion and resting-state functional MRI. To quantify the communication efficiency within and between different functional systems, we proposed two measures called intra- and inter-module efficiency. Based on the module parcellation of functional backbone network, the intra- and inter-module efficiency of SC and FC networks was calculated for each participant. For the SC network, CIS showed decreased inter-module efficiency between the sensory-motor network (SMN), the visual network (VN), the default-mode network (DMN) and the fronto-parietal network (FPN) compared with HC, while MS showed more widespread decreased module efficiency both within and between modules relative to HC and CIS. For the FC network, no differences were found between CIS and HC, and a decreased inter-module efficiency between SMN and FPN and between VN and FPN was identified in MS, compared with HC and CIS. Moreover, both intra- and inter-module efficiency of SC network were correlated with the disability and cognitive scores in MS. Therefore, our results demonstrated early SC changes between modules in CIS, and more widespread SC alterations and inter-module FC changes were observed in MS, which were further associated with cognitive impairment and physical disability.

## Introduction

Clinically isolated syndrome (CIS) is the first manifestation of most MS patients (Noseworthy et al., 2000; Miller et al., 2005). The conversion rate of CIS to MS is highly variable from 8–80% depending on the clinical presentation, MRI features, serum biomarkers and follow up period (Miller et al., 2012). Many previous studies have demonstrated brain atrophy (Charil et al., 2007; Barkhof et al., 2011), diffusion abnormalities (Ciccarelli et al., 2008; Rovaris et al., 2008; Dineen et al., 2009; Liu et al., 2012a), or functional alterations (Roosendaal et al., 2010; Hawellek et al., 2011; Rocca et al., 2012; Tomassini et al., 2012) in CIS and MS. However, the brain is an integrative complex network that cannot be fully understood without proper knowledge of the brain’s topology (Bullmore and Sporns, 2012; Stam and van Straaten, 2012).

Both structural and functional network studies have demonstrated altered network metrics such as decreased global and local efficiency in MS (He et al., 2009a; Shu et al., 2011; Liu et al., 2016), and its relationship with clinical disability (Shu et al., 2011; Schoonheim et al., 2013). However, it is still unknown how network changes on a functional level relate to a structural level for MS, highlighting the need for combined functional and structural network studies. Furthermore, previous network studies have mainly focused on global and regional topological properties (He et al., 2009a; Shu et al., 2011; Schoonheim et al., 2013; Rocca et al., 2014; Tewarie et al., 2015). The brain networks have also been shown to be highly modularized (Bullmore and Sporns, 2009; He et al., 2009b). Detection and characterization of modular structure in the brain system can help us to identify groups of anatomically and/or functionally associated components. Alterations in the modular organization of the brain in CIS and different MS subtypes have been also shown for structural (Kocevar et al., 2016; Muthuraman et al., 2016) and functional networks (Gamboa et al., 2014), even with longitudinal approaches (Fleischer et al., 2017). These findings suggested module-specific topological properties may be more sensitive and specific than global and regional properties to reflect brain alterations and predict the clinical disability in CIS and MS.

Thus, the aim of the present study using diffusion and rs-fMRI techniques combined with graph theoretical analysis was to investigate: (i) whether the module efficiency of structural and functional networks is affected in CIS, (ii) the differences of structural and functional modules between CIS and MS, and (iii) the relationship between structural and functional alterations and their association with clinical variables.

## Materials and Methods

### Participants

We recruited 41 CIS patients (optic neuritis, *n* = 18; spinal cord syndrome, *n* = 16; brainstem syndrome, *n* = 5; cerebellar syndrome, *n* = 2), 32 relapsing-remitting MS patients and 35 healthy controls (HC). All CIS patients were prospectively examined within 6 months from onset with a single clinical episode suggestive of MS (Miller et al., 2012) before steroid treatment. Fifteen CIS patients fulfilled the 2010 modified McDonald Criteria in space dissemination, while none fulfilled the time dissemination. Other patients (*n* = 26) presented with normal brain MRI. All RRMS patients were diagnosed according to the 2010 modified McDonald Criteria (Polman et al., 2011) and required to be relapse-free and without treatment with disease-modifying medications or steroids in the 4 weeks prior to MRI scanning. All of the participants were right-handed, as measured by the Edinburgh Inventory (Oldfield, 1971). The study was approved by the local Institutional Medical Ethics Committee and all participants gave written informed consent.

### Clinical Evaluation

The main demographic and clinical characteristics (**Table 1**), including the Expanded Disability Status Scale (EDSS) score (Kurtzke, 1983), the disease duration, the MMSE and the Paced Auditory Serial Addition Test (PASAT2 and PASAT3 versions) were assessed by an experienced neurologist (HD).

**Table 1:** The demographic information and clinical characteristics of all participants.

	Controls (*n* = 35)	CIS (*n* = 41)	MS (*n* = 32)	F/T/χ^2^/Z value	*P* value
Mean Age (years)	35.0 ± 11.5	35.7 ± 10.7	34.8 ± 8.3	0.08#	0.92#
Gender (F/M)	23/12	26/15	24/8	1.17^‡^	0.56^‡^
Mean MMSE	29.1 ± 1.3	27.6 ± 1.4	25.9 ± 1.8	36.44#	<0.001#
Mean PASAT2	46.7 ± 9.2	39.4 ± 7.7	35.4 ± 9.8	14.14#	<0.001#
Mean PASAT3	53.9 ± 6.2	47.5 ± 7.6	41.0 ± 8.7	24.38#	<0.001#
Mean disease duration (months)	-	2.6 ± 2.5	41.8 ± 28.7	8.73*	<0.001*
Mean TWMLL (ml)	-	4.5 ± 10.6	10.3 ± 10.4	2.33*	0.023*
Median EDSS (range)	-	2.0 (0–6)	3.5 (0–6.5)	3.35§	< 0.001§

*CIS, clinically isolated syndrome; MS, multiple sclerosis; MMSE, Mini-Mental State Examination; PASAT = Paced Auditory Serial Addition Test; EDSS, Expanded Disability Status Scale; TWMLL, Total White Matter Lesion Load. Data are mean ± standard deviation, except for EDSS presented in median (range). ^#^*P*-value and *F*-value were obtained by one-way analysis of variance among three groups. ^‡^*P*-value and χ^*2*^ value were obtained by χ^*2*^ test among three groups. ^∗^*P*-value and *T*-value were obtained by two-sample *t*-test between CIS and MS groups. ^§^*P*-value and *Z*-value were obtained by Wilcoxon rank sum test between CIS and MS groups.*

### MRI Data Acquisition

The MRI data was acquired using a SIEMENS TRIO 3T scanner in the Department of Radiology, Xuanwu Hospital. All participants underwent high-quality MRI scanning, which included a 3D T1-weighted MRI scan [176 sagittal slices, slice thickness = 1 mm, repetition time (TR) = 1600 ms, echo time (TE) = 2.13 ms, field of view (FOV) = 224 mm × 256 mm, acquisition matrix = 224 × 256], a T2-weighted MRI scan (35 axial slices, slice thickness = 4 mm, TR = 5000 ms, TE = 87 ms, FOV = 256 mm× 256 mm, acquisition matrix = 256 × 256), a DTI scan (60 axial slices, slice thickness = 2 mm, 30 diffusion directions with *b* = 1000 s/mm^2^, and an additional b0 image, TR = 11000 ms, TE = 98 ms, FOV = 256 mm × 256 mm, acquisition matrix = 128 × 128, average = 2) and a rs-fMRI scan (40 axial slices, slice thickness = 3 mm, TR = 2000 ms, TE = 30 ms, FOV = 220 mm × 220 mm, acquisition matrix = 64 × 64, 180 image volumes).

### MRI Data Preprocessing

#### DTI Data Preprocessing

The preprocessing procedure for DTI data included eddy current and motion artifact correction, estimation of the diffusion tensor and calculation of the fractional anisotropy (FA). First, the eddy current distortions and the motion artifacts in the DTI data were corrected by applying an affine alignment of the diffusion-weighted images to the b0 images. Accordingly, the *b*-matrix was reoriented based on the transformation matrix. After this process, the diffusion tensors were estimated by solving the Stejskal and Tanner equation (Basser et al., 1994), and the reconstructed tensor matrix was diagonalized to obtain 3 eigenvalues (λ1, λ2, λ3) and their corresponding eigenvectors. The FA value of each voxel was also calculated. The preprocessing of DTI data was performed with the FDT toolbox in FSL^1^.

#### rs-fMRI Data Preprocessing

The preprocessing of rs-fMRI data included motion correction, brain extraction, spatial smoothing, band-pass filtered (0.01 – 0.1 Hz) the data and regressed out nuisance covariates, including six rigid body motion parameters, volumes corresponding to motion spikes, and average WM, cerebrospinal fluid (CSF), and global time series. The first 10 functional volumes were discarded to allow for stabilization of the initial signal and adaptation of the participants to the circumstances. The preprocessing of rs-fMRI data was performed with SPM8^2^ and DPARSF software^3^ (Yan and Zang, 2010).

#### Measurement of WM Lesion Load

Hyperintense white matter (WM) lesions of each patient were manually delineated on the T2-weighted images by an experienced radiologist (YL) who was blind to the clinical details using MRIcro software^4^. Then the total WM lesion load (TWMLL) for each patient was calculated.

### Network Construction

Nodes and edges are the two fundamental elements of a network. In this study, we constructed individual structural and functional connectomes using the following procedures.

#### Network Node Definition

The Automated Anatomical Labeling (AAL) template (Tzourio-Mazoyer et al., 2002) was used to define the network nodes. Briefly, individual T1-weighted images were coregistered to the b0 images in the DTI space. The transformed T1 images were segmented into gray matter (GM), WM and CSF, and then non-linearly transformed to the ICBM152 T1 template in the MNI space. The inverse transformations were used to warp the AAL atlas from the MNI space to the DTI native space. Using this procedure, we obtained 90 cortical and subcortical ROIs (45 for each hemisphere, see **Table 2**), each representing a node of the network. To ensure the consistency of brain parcellation maps, all rs-fMRI images were also coregistered with b0 image. The results of coregistration were visually checked for each participant by an experienced neuroscientist (NS) with 10 years experience in image analysis. All the procedure was performed using SPM8 software.

**Table 2:** Cortical and subcortical regions of interest defined in the study.

Index	Regions	Abbreviations	Index	Regions	Abbreviations
(1,2)	Precental gyrus	PreCG	(47,48)	Lingual gyrus	LING
(3,4)	Superior frontal gyrus, dorsolateral	SFGdor	(49,50)	Superior occipital gyrus	SOG
(5,6)	Superior frontal gyrus, orbital part	ORBsup	(51,52)	Middle occipital gyrus	MOG
(7,8)	Middle frontal gyrus	MFG	(53,54)	Inferior occipital gyrus	IOG
(9,10)	Middle frontal gyrus, orbital part	ORBmid	(55,56)	Fusiform gyrus	FFG
(11,12)	Inferior frontal gyrus, opercular part	IFGoperc	(57,58)	Postcentral gyrus	PoCG
(13,14)	Inferior frontal gyrus, triangular part	IFGtriang	(59,60)	Superior parietal gyrus	SPG
(15,16)	Inferior frontal gyrus, orbital part	ORBinf	(61,62)	Inferior parietal, but supramarginal and angular gyri	IPL
(17,18)	Rolandic operculum	ROL	(63,64)	Supramarginal gyrus	SMG
(19,20)	Supplementary motor area	SMA	(65,66)	Angular gyrus	ANG
(21,22)	Olfactory cortex	OLF	(67,68)	Precuneus	PCUN
(23,24)	Superior frontal gyrus, medial	SFGmed	(69,70)	Paracentral lobule	PCL
(25,26)	Superior frontal gyrus, medial orbital	ORBsupmed	(71,72)	Caudate nucleus	CAU
(27,28)	Gyrus rectus	REC	(73,74)	Lenticular nucleus, putamen	PUT
(29,30)	Insula	INS	(75,76)	Lenticular nucleus, pallidum	PAL
(31,32)	Anterior cingulate and paracingulate gyri	ACG	(77,78)	Thalamus	THA
(33,34)	Median cingulate and paracingulate gyri	DCG	(79,80)	Heschl gyrus	HES
(35,36)	Posterior cingulate gyrus	PCG	(81,82)	Superior temporal gyrus	STG
(37,38)	Hippocampus	HIP	(83,84)	Temporal pole: superior temporal gyrus	TPOsup
(39,40)	Parahippocampal gyrus	PHG	(85,86)	Middle temporal gyrus	MTG
(41,42)	Amygdala	AMYG	(87,88)	Temporal pole: middle temporal gyrus	TPOmid
(43,44)	Calcarine fissure and surrounding cortex	CAL	(89,90)	Inferior temporal gyrus	ITG
(45,46)	Cuneus	CUN			

*The regions are listed in terms of a prior template of an AAL-atlas (Tzourio-Mazoyer et al., 2002).*

#### Structural Connectome

**FIGURE 1:**
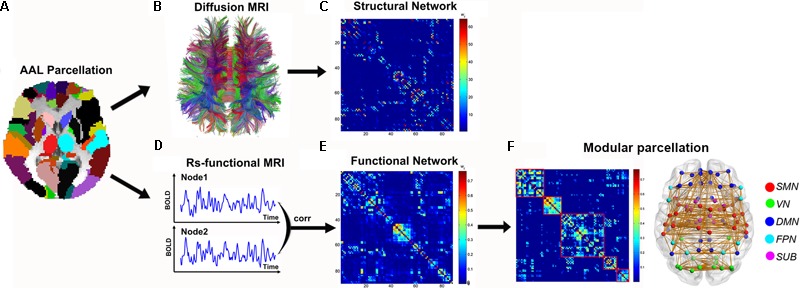
The flowchart of structural and functional connectome construction. **(A)** Individual T1 images and AAL template were used for automatic parcellation of the cortex into 90 brain regions, forming the nodes of the individual brain networks. **(B)** Streamline tractography was applied to the DTI data to reconstruct the white matter pathways. From the set of reconstructed streamlines, the streamlines that interconnected regions *i* and *j* from the set of 90 regions were taken as an edge between nodes *i* and *j* in the structural brain network. The streamline count was taken to represent the weight of the connection and was aggregated into a structural connectivity (SC) matrix **(C)**. **(D)** Functional connectivity (FC) between nodes *i* and *j* was computed as the level of correlation between their rs-fMRI and blood oxygenation level dependent (BOLD) time series, resulting in a matrix, FC **(E)**. Module parcellation was performed based on the FC backbone to parcellate the brain network into different modules **(F)**. For details, see the Section “Materials and Methods.”

Diffusion MRI tractography was performed to reconstruct the whole-brain fiber streamlines with Diffusion Toolkit^5^. All the tracts in the dataset were computed by seeding each voxel with an FA greater than 0.2. The tractography was terminated if it turned an angle greater than 45 degrees or reached a voxel with an FA less than 0.2 (Mori et al., 1999). Two brain regions were considered structurally connected if there were at least three fiber streamlines with two end-points located in these two regions (Shu et al., 2011). Then, the number of the interconnecting streamlines between two regions was defined as the weights of the network edges. Therefore, for each participant, a weighted 90 × 90 structural connectivity (SC) network was constructed (**Figure 1**).

#### Functional Connectome

Based on the brain parcellation map and coregistered rs-fMRI images, the Pearson correlation coefficient of mean time-series between any pair of ROIs (within GM voxels) and their corresponding significance levels (i.e., *p*-values) were calculated. For each participant, a weighted 90 × 90 functional connectivity (FC) network was constructed (**Figure 1**). To remove the spurious correlations in the FC networks, a thresholding procedure was used to convert the correlation matrices derived above to sparse, weighted networks. Specifically, we employed network sparsity (S) (defined as the number of existing edges divided by the maximum possible number of edges in a network) as the thresholding measurement in this study. For example, at a sparsity 10%, the strongest 10% correlations were filtered and retained in individual networks while the others were set to 0. Given the lack of a conclusive way to select a single threshold, individual correlation matrices were thresholded over a consecutive sparsity range of 0.05 < S < 0.40 (interval = 0.025). All the above procedures were performed with the GRETNA^6^ (Wang et al., 2015) and DPARSF software (Yan and Zang, 2010).

### Module Parcellation of Functional Brain Connectome

Through modular parcellation, the FC network can be divided into different functional modules composed of regions with similar functions (He et al., 2009b), while not for the SC network. To investigate the SC and FC alterations in specific functional systems, the module parcellation was only applied to functional brain connectome. First, significantly positive FCs were detected by performing a non-parametric one-tailed sign test and were assigned the average edge weight (FC strength) across all participants to combine as a backbone network. Second, based on the FC backbone, module detection was performed with an optimized simulated annealing approach (Newman, 2006) to parcellate the brain network into different modules. Briefly, the aim of this module identification process is to find a specific partition (p) which yields the largest network modularity, Q(p), which quantifies the difference between the number of intra-module links of actual network and that of random network in which connections are linked at random. The modularity Q(p) for a given partition p of the brain network is defined as (Newman and Girvan, 2004):

$$Q(p)=\sum_{S=1}^{N_{m}}\left[\frac{l_{s}}{L}-{\left(\frac{d_{s}}{2L}\right)}^{2}\right]$$

where N_m_ is the number of modules, L is the number of connections in the network, l_s_ is the number of connections between nodes in module s, and d_s_ is the sum of the degrees of the nodes in module *s*.

### Communication Efficiency Within and Between Modules

Topological efficiency within and between specific functional modules was proposed by us for the first time to quantify the local information processing within modules and the information exchange between modules, respectively. If each module was considered as a subgraph, the intra-module efficiency measures the efficiency of information processing within this subgraph, which reflects the extent of functional segregation within different modules. Inter-module efficiency reflects the efficiency of information exchange between different modules, which reflects the extent of functional integration across modules.

#### Intra-Module Efficiency

The intra-module efficiency measures the global efficiency of the parallel information transfer within the same module, which can be computed as follows:

$$Eff(M)=\frac{1}{N(N-1)}\sum_{i\neq j\in M}^{}\frac{1}{L_{ij}}$$

where N is the number of nodes in module M, i and j are the nodes in module M, and L_ij_ is the shortest path length between node i and node j within the same module M. The shortest path length, L_ij_, is defined as the sum of the edge lengths (the reciprocal of the edge weight, 1/w_ij_) along the path between node *i* and node *j* with the shortest length.

#### Inter-Module Efficiency

The inter-module efficiency measures the global efficiency of the parallel information transfer between two different modules, which can be computed as follows:

$$Eff(M_{pq})=\frac{1}{N_{pq}(N_{pq}-1)}\sum_{i\in M_{p},j\in M_{q}}^{}\frac{1}{L_{ij}}$$

Where N_pq_ is the total number of nodes in modules M_p_ and M_q_, *i* and *j* are the nodes in modules M_p_ and M_q_ respectively, and L_ij_ is the shortest path length between node *i* and node *j* in two different modules. All code for module efficiency is available upon request.

As the modular structure based on the FC backbone was identified, we applied this modular parcellation into both SC and FC networks, and calculated the intra- and inter-module efficiency for both SC and FC networks individually. For the FC network, the module efficiency was calculated at each sparsity level, resulting in curves or functions of the sparsity threshold. Then, the area under curve (AUC) values of the module efficiency were calculated for each participant.

### Statistical Analysis

Demographic factors, including age and gender, among the three groups were compared using ANOVA or the χ2 test. For the group comparisons of the module efficiency, we performed one-way ANOVA, and *post hoc* pairwise comparisons were performed using two-sample *t*-test if ANOVA yielded significant results (*p* < 0.05, corrected). To investigate the relationship between structural and functional alterations in module efficiency, partial correlation analysis was performed across all patients, while including the group effect as a covariate. Additionally, correlation coefficients between the altered module efficiency and clinical variables (EDSS, disease duration, PASAT and MMSE) were calculated in each patient group. We chose *p* < 0.001 as the threshold of significance to correct for multiple comparisons. All of the above statistical analyses were implemented using the Matlab program (The MathWorks, Inc.).

## Results

### Demographic and Clinical Characteristics

There were no significant differences in age (*p* = 0.92) or gender (*p* = 0.56) among the three groups. As for neuropsychological tests, both patient groups showed lower MMSE, PASAT2 and PASAT3 scores than the HC, and the MS patients exhibited lower MMSE and PASAT scores than the CIS group. Eight CIS patients (8/41; 19.5%) and 19 MS patients (19/32; 59.4%) had abnormal PASAT3 performance, and 3 CIS patients (3/41; 7.3%) and 8 MS patients (8/32; 25.0%) had abnormal PASAT2 performance, by defining ≥ 2SD below the average score of HC as abnormal. In addition, MS patients had larger TWMLLs, longer disease durations and higher EDSS scores than CIS patients (**Table 1**).

### Modular Organization of Functional Brain Connectome

**FIGURE 2:**
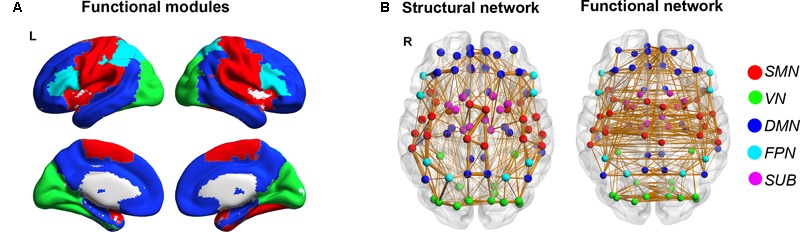
Modular organization of the functional brain connectome. Five functional modules were identified based on the functional backbone network, which are represented in five different colors overlaid on the cortical surface **(A)** and are shown on the backbones of SC and FC networks with different colors in nodes **(B)** (red: SMN; green: VN; blue: DMN; cyan: FPN; magenta: SUB).

A significant modular architecture of the FC backbone network across all participants was identified (Q_max_ = 0.56), separating the brain into five different modules (**Figure 2**). Module I consists of 22 regions mostly from sensory-motor, parietal and temporal cortices, such as bilateral precentral and postcentral gyrus, supplementary motor area, paracentral lobule, supramarginal gyrus, insula, rolandic, superior temporal gyrus and heschl’s gyrus that are mainly associated with the somatosensory, motor and auditory functions (sensory-motor network, SMN). Module II is composed of 14 regions from occipital lobe, including bilateral superior, middle and inferior occipital gyrus, calcarine fissure, cuneus, lingual gyrus and fusiform gyrus that are primarily specialized for visual processing (visual network, VN). Module III includes 36 regions from medial frontal and parietal regions and lateral temporal cortices, such as bilateral superior frontal gyrus, orbital part of middle and inferior frontal gyus, anterior and posterior cingulate cortices, precuneus, angular gyrus, hippocampus and parahippocampal gyrus, middle and inferior temporal gyrus, which are key components of the default-mode network (DMN) (Raichle et al., 2001). Module IV is composed of 8 inferior frontal and parietal regions, including bilateral inferior frontal gyus (both opercular and triangular parts), superior parietal gyrus and inferior parietal lobule that are known to be predominantly involved in attention processing (fronto-parietal network, FPN). The final Module V includes 10 paralimbic and subcortical regions, such as bilateral middle cingulate gyrus, thalamus, caudate, putamen and palladium that are mainly composed of the subcortical system (subcortical network, SUB). Notably, the bilateral homotopic regions were parcellated into the same module. The modular structure of functional brain network is highly consistent with the findings of previous studies (He et al., 2009b).

### Group Differences in Module Efficiency of Structural and Functional Connectomes

**FIGURE 3:**
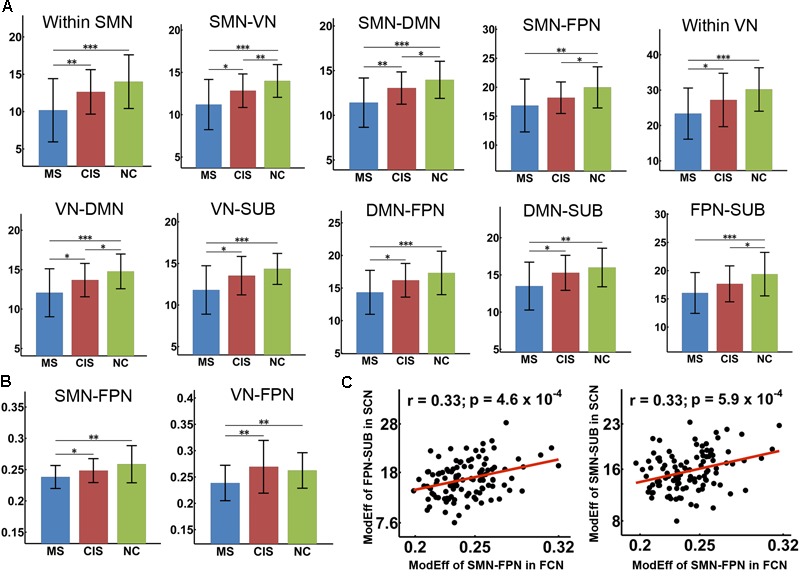
Decreased module efficiency of the SC and FC networks in CIS and MS patients. The bar and error bar represent the mean values and standard deviations of the module efficiency in each group. **(A)** Significantly reduced module efficiency of the SC networks was observed in both CIS and MS patients relative to the controls (all *p* < 0.05, corrected). **(B)** Significantly decreased inter-module efficiency was found in the FC networks in MS patients relative to controls and CIS patients. ^∗^*p* < 0.05; ^∗∗^*p* < 0.01; ^∗∗∗^*p* < 0.005. **(C)** Significant correlations were identified between the decreased module efficiency of the SC network and FC network across all patients (*p* < 0.001). ModEff, module efficiency; FCN, functional connectivity network; SCN, structural connectivity network.

For the module efficiency of the SC network, we found significant group differences within SMN, within VN, between SMN and VN, between SMN and FPN, between FPN and SUB, and between DMN and all other modules (all *p* < 0.05, Bonferroni correction) (**Figure 3A**). A trend of group difference between SMN and SUB (*p* = 0.004) was also identified. *Post hoc* analyses revealed that MS patients had decreased values in all of the above intra- and inter-module efficiency relative to both HC and CIS patients (all *p* < 0.05). Furthermore, the CIS patients showed decreased inter-module efficiencies between SMN and VN (*p* = 0.0088), between SMN and DMN (*p* = 0.025), between SMN and FPN (*p* = 0.014) and between VN and DMN (*p* = 0.017) when compared with controls.

For the module efficiency of the FC network, significant group differences were found between SMN and FPN and between VN and FPN (all *p* < 0.005, uncorrected) (**Figure 3B**). MS patients showed decreased values relative to both HC and CIS patients in all of those inter-module efficiencies. No differences were found between CIS patients and the HC (all *p* > 0.05).

For the relationship between SC and FC alterations across all patients, we found that the decreased inter-module efficiency between SMN and FPN of the FC network was significantly correlated with the decreased inter-module efficiency between FPN and SUB (*r* = 0.33; *p* = 0.0005) and that between SMN and SUB (*r* = 0.33; *p* = 0.0006) of the SC network (**Figure 3C**). For each patient group (CIS or MS), similar correlation results between structural and functional alterations were found.

### Relationship Between Decreased Module Efficiency and Clinical Variables

**FIGURE 4:**
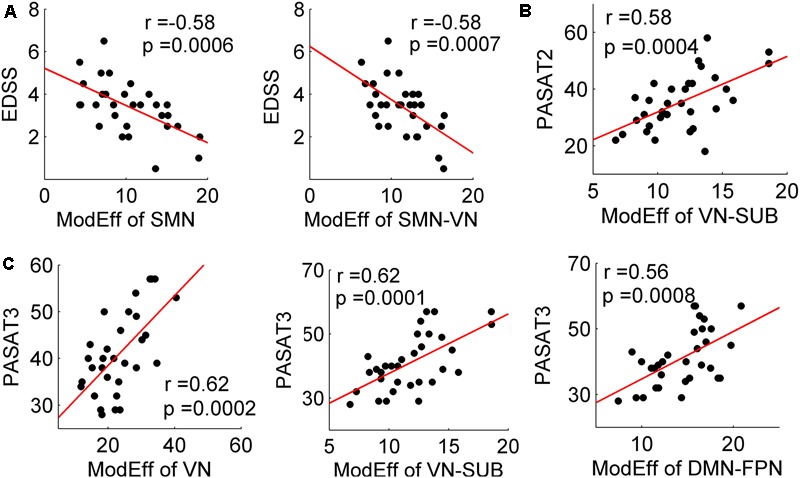
Correlation between decreased module efficiency and clinical variables. Plots showing the linear correlation between altered module efficiency of the SC networks with EDSS **(A)**, PASAT2 **(B)**, and PASAT3 **(C)** scores in MS patients (all *p* < 0.001).

For the MS patients, module efficiency in the SC network was significantly correlated with the EDSS, PASAT2 and PASAT3 scores (all *p* < 0.001) (**Figure 4**). Specifically, increased EDSS scores were correlated with decreased module efficiency of the SC network within SMN (*r* = -0.58; *p* = 0.0006), and between SMN and VN (*r* = -0.58; *p* = 0.0007). The decreased PASAT2 scores were correlated with the decreased module efficiency of the SC network between VN and SUB (*r* = 0.58; *p* = 0.0004). The decreased PASAT3 scores were correlated with the deceased module efficiency of the SC network within VN (*r* = 0.62; *p* = 0.0002), between VN and SUB (*r* = 0.62; *p* = 0.0001), and between DMN and FPN (*r* = 0.56; *p* = 0.0008). No significant correlations were identified between altered module efficiency and disease duration. Furthermore, no correlations with clinical variables were found in the CIS group (all *p* > 0.05).

### Effect of TWMLL on Modular Analysis

To exclude the TWMLL effect on the module analysis, we have repeated the statistical analyses by adding the TWMLL as a covariate. Most of the results remained largely unchanged, but less significance of group differences in the intra- and inter-module efficiency were found, which may be due to part contribution of TWMLL to the group differences in module efficiency. The detailed results of group differences and clinical correlations were as follows:

#### Between-Group Differences

For the module efficiency of the SC network, we found significant group differences within SMN, within VN, between SMN and VN, between VN and SUB, between FPN and SUB, and between DMN and all other modules (all *p* < 0.01, uncorrected). A trend of group difference between SMN and FPN (*p* = 0.02), between SMN and SUB (*p* = 0.03) and within DMN (*p* = 0.03) was also identified. *Post hoc* analyses revealed that MS patients had decreased values in all of the above intra- and inter-module efficiency relative to both HC and CIS patients (all *p* < 0.05). Furthermore, the CIS patients showed decreased inter-module efficiencies between SMN and VN (*p* = 0.01), between SMN and DMN (*p* = 0.04), between SMN and FPN (*p* = 0.03) and between VN and DMN (*p* = 0.03) when compared with controls.

For the module efficiency of the FC network, significant group differences were found between SMN and FPN (*p* = 0.008) and between VN and FPN (*p* = 0.01). MS patients showed decreased values relative to both HC and CIS patients in all of those inter-module efficiencies. No differences were found between CIS patients and the HC (all *p* > 0.05).

#### Clinical Correlations

For the MS patients, module efficiency in the SC network was significantly correlated with the EDSS, PASAT2 and PASAT3 scores (all *p* < 0.05). Specifically, increased EDSS scores were correlated with decreased module efficiency of the SC network within SMN (*r* = -0.40; *p* = 0.030), and between SMN and VN (*r* = -0.42; *p* = 0.023). The decreased PASAT2 scores were correlated with the decreased module efficiency of the SC network between VN and SUB (*r* = 0.61; *p* = 0.0004). The decreased PASAT3 scores were correlated with the deceased module efficiency of the SC network within VN (*r* = 0.62; *p* = 0.0003), between VN and SUB (*r* = 0.61; *p* = 0.0004), and between DMN and FPN (*r* = 0.52; *p* = 0.003). No correlations were found in the CIS group (all *p* > 0.05).

## Discussion

In the present study, we investigated the alterations in the module efficiencies of structural and functional networks in CIS and MS patients by combining DTI and rs-fMRI with graph theoretical approaches. Our results demonstrated the following: (i) at the earliest stage of MS (CIS), structural network changes were mainly located in inter-modules without significant functional network alterations; (ii) more widespread and severe alterations in structural and functional networks were observed in MS; (iii) the correlation between decreased SC and FC connections were mediated by a subcortical network; and (iv) the reduced module efficiency of structural networks was associated with cognitive impairment and physical disability in MS.

Recent brain network studies have consistently revealed that the modular structure is a non-trivial property of the brain connectome, which supports for the functional specialization and segregation of the human brain (Bullmore and Sporns, 2009). By detecting the modules of the functional connectome, we parcellated the brain into different functional systems, which are comparable with previously reported modular decompositions of rs-fMRI networks (He et al., 2009b; Wang et al., 2013). Importantly, the concept of module efficiency was proposed here, which is used to quantify the communication efficiency within and between different functional systems based on the structural or functional connectivity network. The loss or reorganization of the SC or FC in disease populations can be detected by the alterations of the module efficiency, and can be specified to different functional systems.

In the earliest stage of MS (CIS), the predominant decreased module efficiency of SC networks is located between modules of the SMN, VN, DMN and FPN, which indicates that a long-distance connection was mainly involved at the earliest stage of disease. This is consistent with the previous findings with long WM fibers, such as the corpus callosum damage in CIS (Ranjeva et al., 2003), inducing structural inter-module disconnection. Various pathological factors such as axonal damage, demyelination and gliosis may contribute to the inter-module disconnection (Ciccarelli et al., 2014). In MS, much widespread and severe SC reduction was identified, especially in intra-module connections. The intra-module disconnection is mainly caused by short WM fiber damage or cortical demyelination. This result revealed the dynamic changes of the structural network from inter-modules to widespread changes, including both inter- and intra-modules with disease progression.

No significant alterations in the FC network were identified in CIS, implying that the functions were relatively preserved in CIS although structural disconnections were observed. For MS, inter-module disruptions between the SMN, VN and FPN of the FC network were identified, and SC changes were also observed in these inter-module connections. This finding supports the notion that SC damage may precede functional changes and need to achieve a threshold to cause functional impairment. According to previous studies with controversial findings in CIS and MS patients, decreased or increased functional connectivity or regional spontaneous activity was identified (Roosendaal et al., 2010; Bonavita et al., 2011; Hawellek et al., 2011; Liu et al., 2011, 2012b; Rocca et al., 2012). However, most of these studies used independent component analysis (ICA) or amplitude of low frequency fluctuation (ALFF) methods; our finding using a graph analysis approach suggested decreased FC in both CIS and MS patients from system level, which reflect impaired information processing within and between modules. The different analysis methods (different aspects of brain functional alterations) or the heterogeneity of patients (cognitively preserved or impaired, different disease phases, etc.) may help explain the discrepancies among the different studies.

The widespread structural network changes, in contrast to the relatively mild/limited functional network changes, indicate the partial disassociation between the structural damage and functional impairment/reorganization observed in MS patients. As suggested by previous studies, the relationship between structural and functional connectivity in the healthy human brain is complicated and is not a simply one-to-one correspondence (Honey et al., 2010; Hermundstad et al., 2013; Goni et al., 2014). In disease states, the relationship between brain structure and function may be more complicated. Quantitative analysis revealed that decreased FCs of the SMN-FPN are correlated with reduced SCs of the SMN-SUB and the FPN-SUB in patients, highlighting the subcortical network as a key mediator for structural and functional association, and suggesting the possible structural substrate of functional deficits. Subcortical structures, such as the thalamus, play an important role in signal relay and regulation to the cerebral cortex. Additionally, subcortical damage has been well demonstrated in MS patients (Liu et al., 2015; Tewarie et al., 2015). The relationship between indirect structural connections between the SMN and FPN through the subcortical structures and the functional synchronization between regions of the SMN and FPN should be further studied.

Importantly, both intra- and inter-module efficiencies of SC networks were associated with cognitive scores in MS, suggesting that the SC module efficiency may provide potential biomarkers for assessing and monitoring cognitive impairment in patients. SC module efficiency especially in the SMN module, also showed a moderate correlation with EDSS, indicating that SC networks should also be assessed to evaluate physical disability in MS. A clinical correlation was observed in module efficiency of SC but not FC network, implying that SC is a more robust biomarker for clinical deficits. No significant correlations between module efficiency and clinical variables were observed in CIS patients, implying the module changes in CIS may represent a transitional phase, which need to be validated by a large sample study.

Several limitations should be addressed. First, the samples were obtained from a cross-sectional design, whereas future studies with longitudinal MRI data will be required to validate the findings. Second, deterministic tractography was used for the reconstruction of WM tracts, which may result in the loss of existing fibers due to WM lesions in the patients or the “fiber crossing” problem (Mori and van Zijl, 2002). Future studies should employ more advanced tractography techniques to define the network edges. Third, we only used the anatomical AAL atlas to define network nodes, the test-retest validity of our findings should be examined with some other parcellation schemes with more precise anatomical or functional boundary of the brain regions and more even regional size. Finally, the cognitive assessment only includes PASAT in the current study; comprehensive neuropsychological tests for CIS and MS patients should be examined to evaluate the relationship between alterations of module efficiency and cognitive impairment in different cognitive domains in future studies.

## Conclusion

Our results demonstrated early structural network changes between modules without functional alterations were identified in CIS patients, while more widespread and severe alterations in structural networks and disrupted inter-module efficiency in functional networks were observed in MS patients. The structural network changes were associated with cognitive impairment and physical disability.

## Ethics Statement

This study was carried out in accordance with the recommendations of institutional review board of Xuanwu Hospital with written informed consent from all subjects. All subjects gave written informed consent in accordance with the Declaration of Helsinki. The protocol was approved by the institutional review board of Xuanwu Hospital.

## Author Contributions

YL, NS, and KL: guarantor of integrity of the entire study. YL and NS: study concepts, study design, definition of intellectual content, data analysis, statistical analysis, and manuscript preparation. YL, YD, and NS: literature research. HD and YD: clinical studies. HD, YL, and YD: experimental studies. YD and YL: data acquisition. YL, NS, FB, and KL: manuscript editing. NS, FB, and KL: manuscript review.

## Conflict of Interest Statement

FB serves as a consultant for Bayer-Schering Pharma, Sanofi-Aventis, Biogen Idec, Teva, Merck Serono, Novartis, Roche, Synthon, and Jansen Research. The other authors declare that the research was conducted in the absence of any commercial or financial relationships that could be construed as a potential conflict of interest. The reviewer GG-E and handling Editor declared their shared affiliation, and the handling Editor states that the process nevertheless met the standards of a fair and objective review.

## Footnotes

1 http://www.fmrib.ox.ac.uk/fsl

2 http://www.fil.ion.ucl.ac.uk/spm/software/spm8

3 http://restfmri.net/forum/index.php

4 http://www.mccauslandcenter.sc.edu/mricro/mricro/mricro.html

5 http://www.trackvis.org/dtk

6 http://www.nitrc.org/projects/gretna/

## Abbreviations

CIS: clinically isolated syndrome
DTI: diffusion tensor imaging
EDSS: Expanded Disability Status Scale
MMSE: Mini-Mental State Examination
MRI: magnetic resonance imaging
MS: multiple sclerosis
PASAT: Paced Auditory Serial Addition Test
rs-fMRI: resting-state functional MRI
